# Effect of Unit Cell Type and Pore Size on Porosity and Mechanical Behavior of Additively Manufactured Ti6Al4V Scaffolds

**DOI:** 10.3390/ma11122402

**Published:** 2018-11-28

**Authors:** Haizum Aimi Zaharin, Ahmad Majdi Abdul Rani, Farooq I. Azam, Turnad Lenggo Ginta, Nabihah Sallih, Azlan Ahmad, Nurul Azhani Yunus, Tun Zainal Azni Zulkifli

**Affiliations:** 1 Advance Biomedical Materials and Manufacturing (ABMM), Universiti Teknologi PETRONAS, Seri Iskandar 32610, Malaysia; majdi@utp.edu.my (A.M.A.R.); farooq.i_g03648@utp.edu.my (F.I.A.); turnad.ginta@utp.edu.my (T.L.G.); nabihah.sallih@utp.edu.my (N.S.); azlan.ahmad@utp.edu.my (A.A.); azhani.yunus@utp.edu.my (N.A.Y.); zainal.zulkifli@utp.edu.my (T.Z.A.Z.); 2 Department of Mechanical Engineering, Universiti Teknologi PETRONAS, Seri Iskandar 32610, Malaysia; 3 Department of Electrical Engineering, Universiti Teknologi PETRONAS, Seri Iskandar 32610, Malaysia

**Keywords:** porous, cube, gyroid, selective laser melting, stress shielding effect, Young’s modulus, Ti6Al4V

## Abstract

Porous metal structures have emerged as a promising solution in repairing and replacing damaged bone in biomedical applications. With the advent of additive manufacturing technology, fabrication of porous scaffold architecture of different unit cell types with desired parameters can replicate the biomechanical properties of the natural bone, thereby overcoming the issues, such as stress shielding effect, to avoid implant failure. The purpose of this research was to investigate the influence of cube and gyroid unit cell types, with pore size ranging from 300 to 600 µm, on porosity and mechanical behavior of titanium alloy (Ti6Al4V) scaffolds. Scaffold samples were modeled and analyzed using finite element analysis (FEA) following the ISO standard (ISO 13314). Selective laser melting (SLM) process was used to manufacture five samples of each type. Morphological characterization of samples was performed through micro CT Scan system and the samples were later subjected to compression testing to assess the mechanical behavior of scaffolds. Numerical and experimental analysis of samples show porosity greater than 50% for all types, which is in agreement with desired porosity range of natural bone. Mechanical properties of samples depict that values of elastic modulus and yield strength decreases with increase in porosity, with elastic modulus reduced up to 3 GPa and yield strength decreased to 7 MPa. However, while comparing with natural bone properties, only cube and gyroid structure with pore size 300 µm falls under the category of giving similar properties to that of natural bone. Analysis of porous scaffolds show promising results for application in orthopedic implants. Application of optimum scaffold structures to implants can reduce the premature failure of implants and increase the reliability of prosthetics.

## 1. Introduction

Porous structure is a universal term used to represent the porosity, pore size, pore size distribution and pore morphology of a porous material. Porous structures can be found in form of artificial solid such as porous metal, porous ceramics and polymer foams and also in nature, for example bones, limbs, plant leaves, wood, sponge and coral. Nowadays, porous structures are largely being used in many applications including aerospace, automotive industries and orthopedics. Porous structures can be classified according to their porosity types (closed pores and open pores) and their unit cell arrangement (stochastic and non-stochastic). The unit cells which are built in irregular order of arrangement such as bone it is known as stochastic while the unit cells which are built in regular form such as honeycomb and cube is known non-stochastic. Morphology of porous structures can be analyzed using number of methods that can quantify the build quality. Among these techniques, micro CT provides 3D measurements of pore size, porosity and strut size of porous structure with better accuracy [1].

In biomedical applications, especially for artificial implants, porous structures have shown promising characteristic as they provide a high surface area for bone ingrowth. The combination of suitable porous material, porous architecture and optimum parameters in load-bearing implant reduces the implant stiffness, which overcomes stress shielding effect, preventing aseptic loosening of implant. Selection of optimum morphological parameters such as pore size and porosity are also crucial to ensure successful rate of bone ingrowth [2]. Porous structure with connected surface and appropriate pore size can provide optimum conditions for new bone and capillary formation, improving the osteogenic characteristics of implant [3]. Optimum porosity should be more than 50% for ideal osteointegration, whereas pore size should be in range of 100–700 µm to avoid pore occlusion and to provide sufficient surface area for cell adhesion and increase load bearing capacity [2,4,5]. Appropriate porous architecture also influences the mechanical properties of implant. For instance, Sogutlu et al. [6] developed a method for modeling stochastic architecture which directly replicates bone structure at different region, an alternative solution to mimic the bone geometry, giving more similar mechanical characteristic to the bone. However, Cansizoglu et al. [7] observed that stochastic porous design scaffold shows deformation due to random disconnected nodes of internal structure. On the other hand, higher mechanical properties have been found for non-stochastic lattice structures [8]. Mechanical properties are also a function of the orientation of the structure. Volker et al. [9] studied the influence orientation of struts and microstructure on mechanical behavior of porous structures. Liu et al. [10] studied the mechanism of energy absorption in porous structures designs and optimized the structure topology for balance between bending and compressive strength. Recent studies for biomedical implant application mostly focus on three-dimensional, open cell, non-stochastic cellular structure. Extensive research has been done with cubical structure, owing to the simplest geometrical design with promising results [11,12,13,14]. Regular strut dimension on each vertex of cube geometry generates consistent stress distribution on every strut. Other porous structures, for instance, diamond [13,15,16,17,18], truncated cube [18], truncated cuboctahedron [13,18], tetrahedron [2], rhombicuboctahedron [18] and octet truss [2], have also been studied for orthopedic application. Lately, with unique mechanical and biological behavior, triply periodic minimal surfaces (TPMS) porous design have become the focus of research [19,20,21]. This is due to zero mean curvature, which shows the same character as trabecular bone. Furthermore, the application of gyroid design on variety of materials presents low density character by maintaining high tensile strength, similar to graphene [22]. Gyroid structure has also been observed in the polymer phase diagram located between lamellar and cylindrical phases and it has also been applied experimentally to supercapacitors [23], nano-porous membranes [24] and solar cells [25]. In another study, gyroid structure has been discovered in biological structural coloration of butterfly wing scale in biomimetic materials research [26]. Jason et al. [21] developed an algorithm for constructing gyroid-type porous geometry to facilitate engineers and clinicians in manipulating porous scaffold parameters. Yan et al. [27] evaluated TPMS mechanical properties of manufactured gyroid by Selective Laser Melting (SLM) and concluded that Ti-6Al-4V TPMS scaffold can be customized to replicate human bones and avoid stress shielding on implants, thus increasing durability of implants. 

The selection of porous material for medical implants is also a vital subject considering the functions and type of implants. For load-bearing application, such as hip and knee implants, fabrication requires metallic biomaterial that provides a non-toxic, non-irritant, non-allergenic and non-carcinogenic environment to human body. At the same time, it must offer good response between host bone and implant by promoting osteoinduction, osteoconduction and osteointegration [28]. Examples of biomaterial for joint implant include stainless steel, cobalt–chromium (Co–Cr) alloys and titanium alloys. Monroy et al. [29] studied the pore formation on Co-Cr-Mo alloys, claiming that these alloys are commonly used in biomedical applications as they are known as the hardest biocompatible alloy along with good tensile and fatigue properties. Stainless steel (316L) is a widely used biomaterial as it is strong and cheap. Both stainless steel and Co-Cr alloys depend on the existence of chromium percentage in alloy for their corrosion resistance characteristic [30]. Amongst the materials available for biomedical implants application, Ti-based alloys remain as the popular choice for hard tissue replacement due to their unique microstructure, mechanical, corrosion and resistance properties [31,32]. Titanium alloy porous structures can offer the combination of high compressive strength with low Young’s modulus value as well as higher fatigue strength, suitable for biomedical implants [33,34]. In comparison with other bio-compatible materials for the Young’s Modulus value, Co-Cr alloy and 316L stainless steel show 210–253 GPa and 190–210 GPa, respectively, while the Young’s Modulus of Ti-based alloys is only 100–140 GPa. It is also reported that elements such as Co and Cr have toxic effects from artificial implant to human body [31], whereas Ti-based alloys have many benefits that include lower elastic modulus, excellent corrosion resistance and enhanced biocompatibility [35]. Although Ti-alloys offers low elastic modulus for implant application, the value is still much higher than the Young’s modulus of human bone tissues (4–30 GPa) [36]. This demands adequate morphological alteration of pores to imitate mechanical properties of bone tissues [15]. 

With the advent of additive manufacturing (AM) technology, it is possible for an implant to be manufactured with customized periodic open cellular structure in pre-defined dimension. This technology is expeditiously developing, giving the benefit in controllability on material properties and internal geometry, resulting in promising mechanical and biological response to natural bone. Selective Laser Melting (SLM) is a form of AM that bind powder particles such as titanium, stainless steel and cobalt–chromium with highly powered laser to become three-dimensional solid structure [37]. SLM maximizes the utilization of material since un-melted powder can be re-used for fabrication [38]. It is an accurate and fast manufacturing process compared to other AM technologies and is able to achieve approximately 100% density, producing stronger part and eliminating the postprocessing constraints such as infiltration [39]. Additionally, with post heat treatment on SLM fabricated parts, fatigue life is increased, and energy absorption is enhanced by four-point bending test [40]. Electron beam melting (EBM) is another metal additive manufacturing technique that is becoming popular for manufacturing of bulk and porous structures. As compared to SLM, EBM can produce higher density parts due to vacuum environment [41]. However, fabrication with SLM results in higher compression strength and lower Young’s modulus, which is desirable for implant application [42]. Smith et al. [43] compared the results of analyzed finite element (FE) models with experimental data of compression tested lattice structure, fabricated using SLM technique. They observed lattice structures modeled by beam and brick elements and determined that, by varying the unit cell geometry, the prediction of mechanical properties can be improved. Sun et al. [44] studied the relationship of loading and porosity of porous structure, indicating the importance of fracture load calculation for lattice structures. Processing parameters of manufacturing process also play a vital role on quality and mechanical behavior of porous scaffolds and can be optimized to obtain required properties [45]. Although SLM requires appropriate angle orientation to minimize internal support structure, this process is a reliable technique for manufacturing artificial implants and has been employed to fabricate prostheses that have nearly human-bone-like mechanical properties, with complex open porous structure developed on implant design [12]. In this study, cubic and gyroid scaffold design structures with pore size ranging from 300 to 600 µm were considered. The influences of each scaffold design structure and pore size on porosity and mechanical properties of Ti6Al4V scaffolds were investigated. 

## 2. Materials and Methods

The methodology adopted for this research included visual and structural analysis of porous design structure to predict the mechanical properties and feasibility of structures. The first phase of the methodology involved using numerical simulation tools to study the mechanical behavior of structures. For the second phase, porous samples were fabricated using SLM. Later, samples were used to study the porous design experimentally and to validate the simulation.

### 2.1. Numerical Modeling

**Figure 1:**
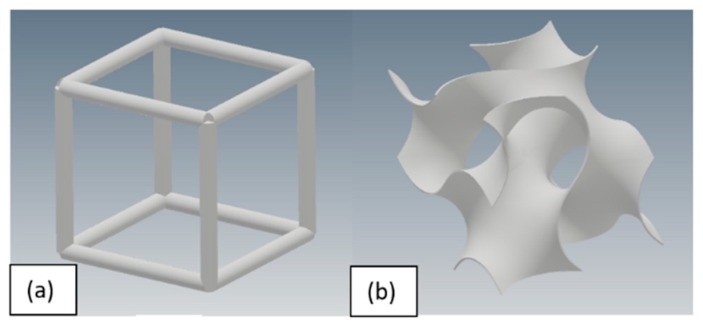
Unit cell architecture: (**a**) cube; and (**b**) gyroid.

Architecture of porous design is crucial as manufacturability and mechanical properties of structure depends on the design. For metallic implants, an important factor effecting the selection of porous design is manufacturing of the structure. Additive manufacturing processes still possess certain limitations which must be considered when selecting the porous design for artificial implants. To study the effect of unit cell type and pore size on porosity and mechanical behavior of additively manufactured Ti6Al4V scaffolds, two different unit cell architectures, namely cube and gyroid, were selected. The selection of these designs was based on the ease in manufacturing of these structures using additive manufacturing. Cube is the simplest porous unit cell design with all the struts at the angle of 90 degrees to each other which make its design easier to manufacture. Gyroid is the unit cell design with no planer surfaces and angle between all the surfaces is approximately 38.1°, less than 45°, adding advantage for gyroid fabrication using additive manufacturing. Unit cells of selected cube and gyroid are shown in Figure 1.

**Figure 2:**
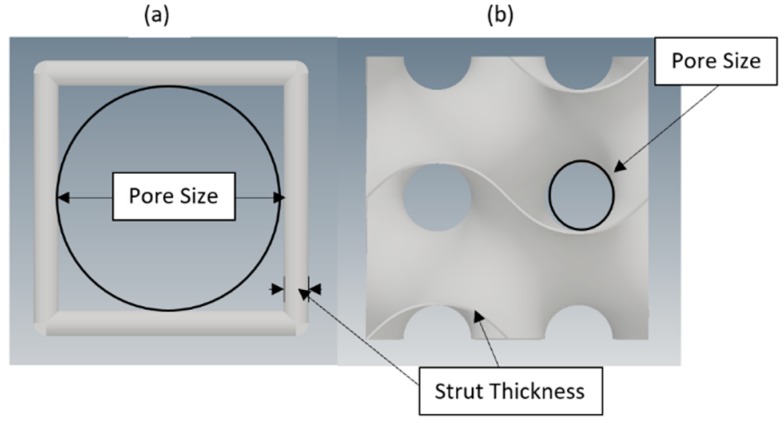
Unit cell pore size and strut thickness for: (**a**) cube; and (**b**) gyroid.

Selection of unit cell architecture for metallic implants depends not only on the mechanical behavior of the design but also on internal parameter of the structure. Criteria for selection involves suitable porosity, pore size and pore interconnectivity, with the goal to achieve satisfactory clinical outcome. Increased porosity of a structure facilitates and benefit bone tissue growth but will also reduce the mechanical strength of structure. Porosity must be greater than 50% to allow bone ingrowth, as mentioned above. Pore size of porous structure has significant impact on bone tissue regeneration. The optimal pore size from various literature is claimed to be 100–700 µm [2,4,5]. Too small pore will hinder bone ingrowth by promoting pore occlusion, whereas too large diameter of pore size will reduce surface area for cell adhesion as well as lessen the load capacity of weight bearing. Thus, a porosity >50% and pore size within the optimal range to sustain good cell migration, reliable mass transportation and safe vascularization besides facilitating sufficient surface area for cells adherence as well as maintaining mechanical stability should be selected. In accordance with these constraints, pore sizes of 300 µm, 400 µm, 500 µm and 600 µm were selected for this research for all designs. Strut thickness for this research was kept constant at 200 µm, corresponding to minimum resolution requirement by manufacturing process. Pore size of unit cell is defined as diameter of the inscribed circle between struts of unit cell in planer view. Strut thickness is the diameter of strut for cube cell type and thickness of periodic surface in gyroid unit cell [13,27]. Pore size and strut thickness for both unit cells are shown in Figure 2.

**Figure 3:**
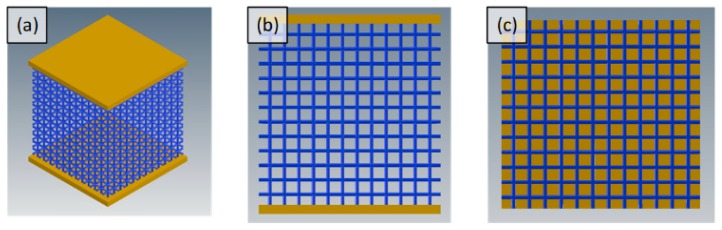
(**a**) Cube sample with plates on top and bottom; (**b**) cube sample from front view; and (**c**) top view of cube porous scaffold without top plate.

**Figure 4:**
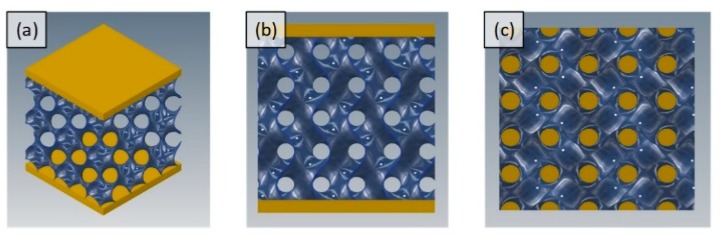
(**a**) Gyroid sample with plates on top and bottom; (**b**) gyroid sample from front view; and (**c**) top view of gyroid porous scaffold without top plate.

After selection of modeling parameters, eight different porous structures were modeled using Autodesk Inventor, including cube and gyroid unit cell designs and their pore size variations. Unit cells of all designs were modeled and patterned in three directions (x, y, z) to get scaffolds of dimension 10 mm × 10 mm × 10 mm. Dimensions of sample are defined by ISO standard (ISO 13314:2011) which requires the length of each side to be 10 mm minimum. This standard was used later for experimental testing of samples. CAD model of scaffolds can be seen in Figure 3 and Figure 4.

After modeling of samples in CAD system (Autodesk Inventor 2017, Autodesk , San Rafael, CA, USA), samples were exported to simulation system for finite element analysis. In this research, titanium alloy Ti6Al4V was selected as a material to be used for samples manufacturing due to its biocompatibility and number of biomedical applications. The same material was used for analysis of samples in simulation software, ANSYS (Release 16.2, ANSYS, Inc., Canonsburg, PA, USA). Since the mechanical properties of Ti6Al4V produced by additive manufacturing has been proven to be different from properties of Ti6Al4V produced by conventional manufacturing, material properties of additively manufactured Ti6Al4V were taken from the literature [15] and added to ANSYS as a new material. The material mechanical properties were applied to each imported sample for analysis. Mechanical properties of materials used for simulation are presented in Table 1.

Porosity of modeled samples was calculated to determine the suitability of design for implants. Porosity was calculated using the following equation.

Porosity = (1 − V/Vs) × 100(1)

where V and Vs are volume of porous structure and volume of solid structure, respectively. Volume of porous samples was taken from CAD software and used to determine the porosity.

After importing the sample model to ANSYS, two plates, on top and bottom, were added to imitate the compression testing method by standard (ISO 13314:2011). For boundary conditions, both top and bottom plates were assumed as rigid body while sample was considered flexible. Remote force on the top plate was applied to each sample, which was later converted to applied stress, whereas bottom plate was fixed to ground. In this study, deformation was considered only in direction of force applied. Directional deformation data were collected, and strain was calculated from deformation. From the simulation, the relationship between porosity and pore size and elastic modulus versus pore size for each sample could be determined. Meshing of analysis model was performed after geometry setup and applying all boundary conditions. Tetrahedron grid was used in this study. Investigation of grid independence was performed using grid with different number of elements.

**Figure 5:**
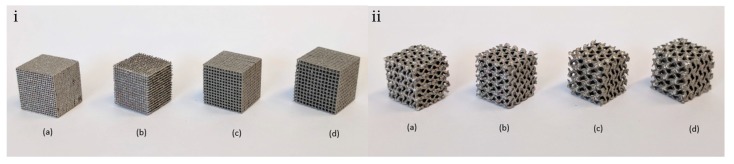
3D Printed (**i**) cube and (**ii**) gyroid samples with pore size: (**a**) 0.3 mm; (**b**) 0.4 mm; (**c**) 0.5 mm; and (**d**) 0.6 mm.

Samples were fabricated using Selective Laser Melting (SLM) process because of its ability to produce complex porous structures as opposed to conventional techniques. Five samples of each type were manufactured, as required by testing standard, which added up to a total of 40 samples. For fabrication process, sample files were imported to pre-processing software of 3D printer using STL file format. EOSINT M250 metal printer was used to print 3D parts, which works on the principle of direct metal laser sintering (DMLS). Designs were pre-processed to select suitable orientation for printing to minimize support structures. Cube samples were printed at angle of 45° to avoid any internal support structure, whereas gyroid samples were printed at normal orientation as all the angles in gyroid geometry are greater than 45°. Support structure at the bottom of parts was used to form base plate, which was later removed in post processing of parts. Grade 23 Titanium alloy (Ti6Al4V) powder was used for manufacturing. Total time taken to print all parts was 48 h. Manufactured samples are shown in Figure 5.

### 2.2. Experiment

Developed samples were subjected to experimental testing for pore size, porosity and structural analysis to validate simulation results. For morphological analysis, printed samples were analyzed to measure the pore size of manufactured porous structure. For this purpose, all samples were passed through microfocus X-Ray CT system. InspeXio SMX-225CT (SHIMADZU Corporation, Tokyo, Japan) was used to acquire CT images of sample’s internal and external structure. 

The porosity of manufactured samples was determined using the same equation to establish theoretical porosity. The equation requires volume of porous sample and volume of solid sample to calculate porosity of sample. Volume of porous sample was calculated using mass, m, per unit density, ρ, relation, as below:

V = m/ρ(2)

All fabricated samples were weighed using weighing scale with 1 mg resolution to get the mass of porous sample. To get the bulk density of material (Ti6Al4V) manufactured by SLM, a solid sample was printed of volume 10 mm^3^. Density of bulk solid was calculated by using the equation above as 4.419 g/cm^3^. Experimental density of material was approximately similar to rated material density by supplier, which is 4.42 g/cm^3^. Calculated value of density and measured value of mass was used in equation to get the volume of porous samples. Values of porous samples were later used in Equations (1) and (2) to get the porosity value.

After visual analysis of produced samples, samples were subjected to compression testing to determine the Young’s modulus and yield strength of porous structure. Compression testing was done following ISO standard (ISO 13314) which is standard testing method for porous and cellular metals. Universal Testing Machine (UTM, GOTECH Testing Machines Inc., Taiwan) was used for the testing with maximum loading capacity of 5 kN. Force was applied with compression speed of 0.01 mm/s and deformation in sample was measured in vertical direction. Measured deformation data were later used for calculation of Young’s modulus and yield strength. 

## 3. Results and Discussion

In this research, properties of samples were analyzed numerically, and then, fabricated samples were subjected to experimental analysis for testing and simulation validation. Comparison of morphological and mechanical properties were made for cubic and gyroid samples for both simulation and experimental results. Results were also compared to properties of natural bone to determine the suitability of porous structures for implant applications.

**Figure 6:**
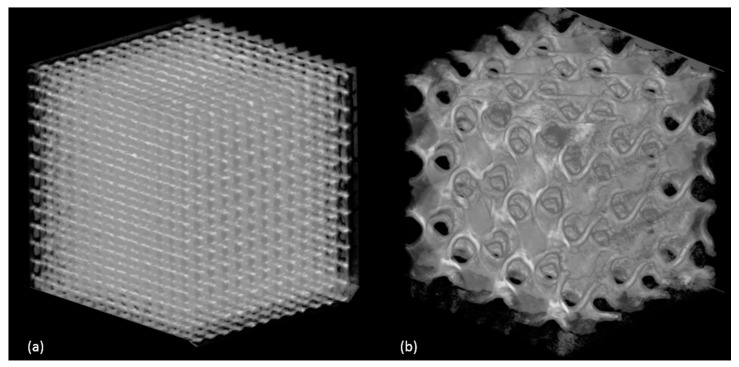
CT reconstruction of samples: (**a**) cube; and (**b**) gyroid.

**Figure 7:**
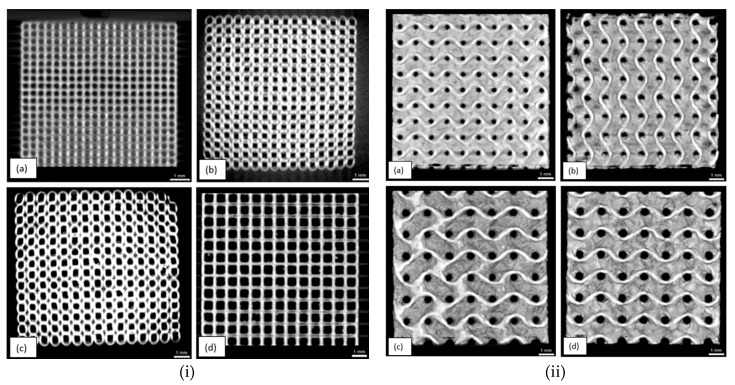
CT Image of (**i**) cube and (**ii**) gyroid sample: (**a**) 0.3 mm; (**b**) 0.4 mm; (**c**) 0.5 mm; and (**d**) 0.6 mm.

Porosity of samples in numerical analysis was calculated using Equation (1), while, for experimental analysis of morphological properties, the percentage of porosity depends on the mass, density and volume of fabricated sample, as explained in Section 2. To avoid pore blockage and allow good permeability, optimum porosity of scaffold samples must be more than 50%. Similar trend of increase in percentage of porosity with increase in pore size is shown in both simulation and experiment. CT analysis of fabricated samples shows some variations in dimensional accuracy. Dimensions of all samples were accurate to design, with less than 0.1% error. However, the pore size and porosity obtained from experimental analysis was lower in value than simulated values. This can be explained with defect in manufacturing process. In SLM, pore size shrinks after solidifying of powder material due to dross formation. This shrinkage effect was not accounted for while manufacturing of samples. Reduction in pore size increases the volume of sample, thus decreasing the porosity of fabricated samples. Manufactured samples were reconstructed and analyzed using X-ray CT system to measure the dimensional accuracy of manufacturing process. Reconstruction images are shown in Figure 6. Pore size of manufactured samples were measured using image analysis software, ImageJ (version 1.52e, National Institutes of Health, Bethesda, MD, USA). CT images of cubic and gyroid porous structures is shown in Figure 7.

**Figure 8:**
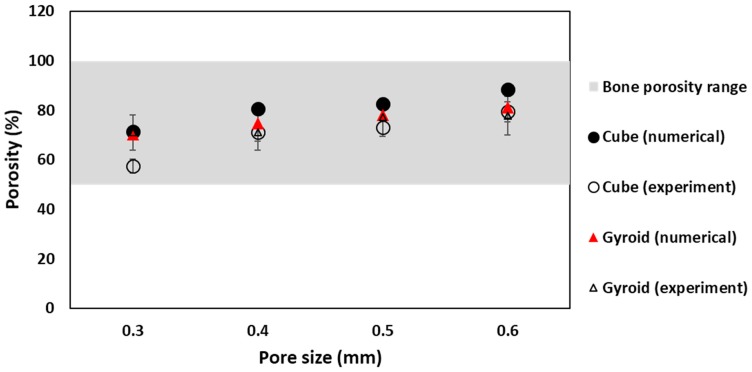
Porosity comparison for cube and gyroid samples in bone porosity range.

For cubical porous structure, the comparison of porosity obtained from simulation and experiment in the range of natural bone requirement is shown in Figure 8. The porosity obtained from experimental analysis is lower in value than simulated values for the reasons explained earlier. Figure 8 clearly shows that, even with the difference in numerical and experimental values, porosity value for cubic samples lies within the range for natural bone porosity value, which makes all cubical scaffold samples suitable for mechanical analysis. For gyroid scaffold samples, the result shows more agreement in its numerical and experimental porosities. Simulated and measured porosity values are approximately similar, with negligible difference. Furthermore, all samples for gyroid show porosity within the range of natural bone, which make them suitable for implant applications. Comparison of numerical, experimental and natural bone porosities for gyroid is shown in Figure 8.

**Figure 9:**
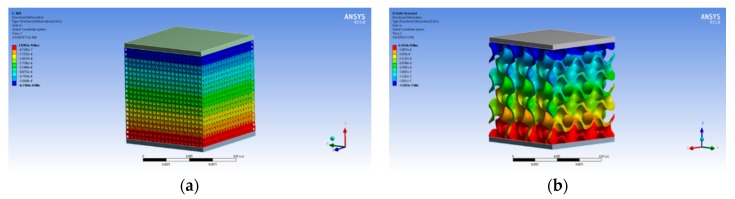
Deformation produced due to applied force in: (**a**) cubic sample; and (**b**) gyroid sample.

For numerical analysis, suitable porous samples are subjected to finite element analysis to calculate their Young’s modulus and yield strength. Forces from 250 N to 4 kN were applied on each sample and the model was solved for deformation in direction of applied force. Figure 9 illustrates deformation produced in sample due to applied force.

Deformation data collected from the analysis were used to calculate strain produced in sample using Equation (3):

ε = (ΔL)/L(3)

where ε is strain, ΔL is the deformation in m and L is the original length, which is 0.01 m. 

Compression stress is calculated from applied force, using Equation (4):

σ = F/A(4)

where σ is the compression stress in Pa, F is the force applied on the sample in N and A is the area of plate, which is 0.0001 m^2^. 

**Figure 10:**
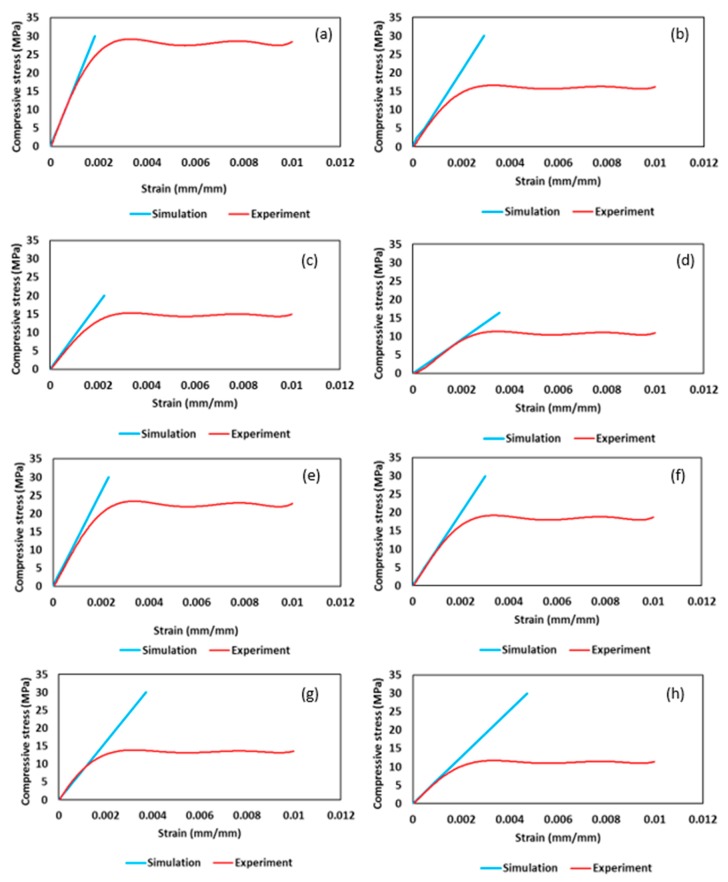
Numerical and experimental stress-strain curve for: (**a**) cube 0.3 mm; (**b**) cube 0.4 mm; (**c**) cube 0.5 mm; (**d**) cube 0.6 mm; (**e**) gyroid 0.3 mm; (**f**) gyroid 0.4 mm; (**g**) gyroid 0.5 mm; and (**h**) gyroid 0.6 mm.

Young’s modulus is calculated using gradient of plotted stress–strain curves. Yield strength is measured from stress–strain graph at strain value of 0.02%. Based on the same pore size range, yield strength also showed decrease in values for cubic and gyroid sample, same as Young’s modulus. Declining trend was observed for yield strength with the growth of pore size. Data obtained from numerical analysis and experimental testing are presented as a graph for compression stress versus strain, plotted for each sample to determine Young’s modulus and yield strength in Figure 10. Numerical stress–strain curve only shows the elastic region of sample, as analysis was performed until elastic limit only. Experimental curve is shown until fracture of first layer. Difference in the values of numerical and experimental can be justified because of manufacturing defects and surface abnormalities. However, the values are in good agreement with each other, with less than 20% relative error.

**Figure 11:**
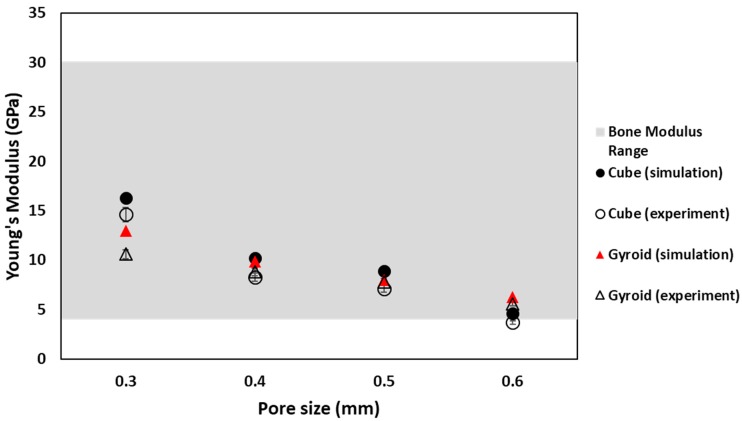
Comparison between numerical and experimental Young’s modulus for cube and gyroid sample in the range of bone modulus.

For an implant to be suitable to replace natural bone, its mechanical properties must also match the mechanical properties of bone. By creating porous scaffold, mechanical strength of materials used for implant application can easily be manipulated. Comparison of elastic modulus of all porous structures was performed with that of natural bone. For cubical sample, simulated and experimental values of Young’s modulus are in good agreement with each other except for cubic sample with pore size 0.3 mm. Cube 0.3 mm shows different behavior because of manufacturing defects. For both cube and gyroid porous samples, elastic modulus values of numerical and experimental analysis depict similar behavior. Young’s modulus decreasing with increasing pore size was due to increasing in sample porosity. In comparison to natural bone modulus value, except cube with 0.6 mm pore size, all cubic samples show modulus value within the natural bone range, making them suitable for artificial implants. For cubic 0.6 mm sample, the experimental value of modulus is lower than the natural bone range, making it unsuitable for artificial implant application. In the case of gyroid porous samples, again, the simulation and experimental values are in better agreement with each other as compared to cubic samples. Gyroid samples also show similar trends with change in pore size as cubic samples. However, for gyroid, all samples give the modulus values within the range of natural bone. Relation of Young’s modulus with pore size for cubic and gyroid porous structure is shown in Figure 11.

**Figure 12:**
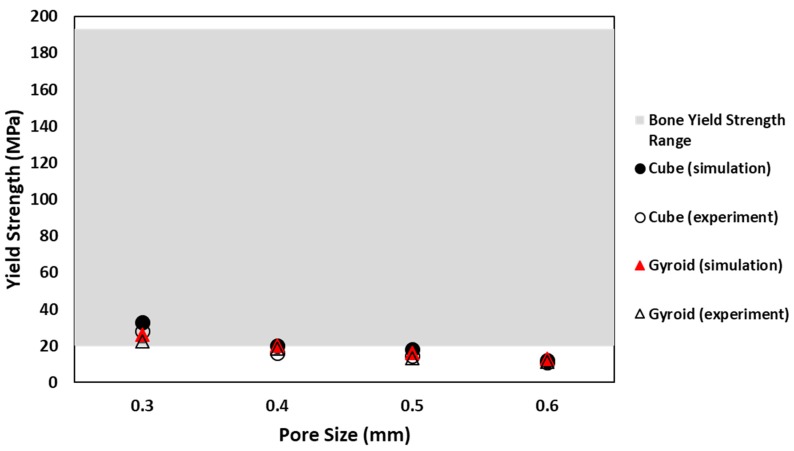
Comparison between numerical and experimental yield strength for cube and gyroid sample in the range of bone yield strength.

Another factor to be considered in selection criteria of suitable porous structure is yield strength of sample. Yield strength value of natural bone reported in the literature is between 20 and 193 MPa [31]. Samples with yield strength values within the range of bone are considered suitable for implants. Numerical and experimental values of both cubic and gyroid samples are in good agreement with each other. In addition, similar trend of decrease in yield strength with increase in pore size is shown by both sample types, for all pore sizes. Comparison of numerical values of yield strength for cubic and gyroid samples shows that only some samples agree with yield value of natural bone. For cubic samples, cube 0.3 mm, cube 0.4 mm and cube 0.5 mm show yield strength value within the range. For gyroid samples, only gyroid with pore sizes 0.3 mm and 0.4 mm agree with yield strength value of bone. However, comparing experimental values of yield strength for cubic and gyroid structure with natural bone, it shows that, except for samples with pore size 0.3 mm, all other samples give yield strength value lower than desired range, which makes them unsuitable to be considered for implant application. Cube 0.3 mm and Gyroid 0.3 mm, with yielding value of 26.1 and 22.4 MPa, respectively, are the only samples that show yield strength with the range of natural bone. Comparison of yield strength for cubic and gyroid structure is shown in Figure 12.

Comparison of all analyzed properties for cube and gyroid porous structures is tabulated in Table 2.

Mechanical properties of implants must lie within the range of bone mechanical properties to avoid stress shielding effect. The elastic modulus and yield strength of cortical bone are 4–30 GPa and 20–190 MPa, respectively [31]. For trabecular bone, the ranges are 0.2–2 GPa and 2–80 MPa, respectively [31]. The elastic modulus and yield strength, for each sample, obtained from analysis are compared with the given range for optimum scaffold. In Table 2, elastic modulus of all samples is within the required range but yield strength of most of the designs is less than the recommended range. Thus, some samples are unsuitable for implant application. Properties of cube with pore size 0.3 mm and gyroid with pore size 0.3 mm are within the specified range of bone. These sample designs are fit to be used as artificial implant for biomedical application. On the other hand, none of the porous structures’ properties lie within the range of trabecular bone properties. To get properties similar to those of trabecular bone, sample structure needs to be more porous to further reduce the elasticity and optimized design should be created to achieve desirable trabecular yield strength. 

Structural analysis of porous designs gives promising results for application of artificial implants that can achieve the properties of natural bone. Implants can be designed using optimum porous scaffold proposed by this study as the internal structure will be extruded to the shape of chosen artificial implant. However, the structure of bone is not uniform and symmetric as in the designed samples. Internal bone structure consists of different parts with variable mechanical properties that make the construction of bone non-uniform and asymmetric. Properties of bone vary in different orientations and parts of bone. Therefore, to produce an implant that mimics bone structure, implant design should consist of various structures with variable porosity. Producing such implant is impossible with conventional manufacturing processes. However, with recent developments in additive manufacturing, porous implants can easily be produced. Additive manufacturing, with its ability to produce complex parts, can produce a part with variable porosity and complex structure. Technologies such as selective laser melting are being used to produce metal parts for several applications. The same technology could also be used to produce artificial implants that are similar to bone structure and properties, which is still a drawback for conventional technology.

Samples analyzed in this research are the first step in development towards producing better implants. Cube or gyroid, all of these structures can be produced by selective laser melting (SLM) without support structures, keeping in view the limitation of metal additive manufacturing. In addition, these structures show optimum properties, with suitable pore size, to form the basis of porous implants. Analysis of these structure has shown reduction in mechanical properties as well as strength, which, when applied to implant application, will reduce the stress shielding effect. Use of these porous architectures will increase the life of prostheses, making them more durable and suitable for human body. 

## 4. Conclusions

In this research, two different porous structure designs were analyzed for their morphological and mechanical properties. Application of porous scaffold to artificial implants is proposed to reduce the problem of stress shielding effect. From the results, it can be concluded that increase in pore size of porous scaffold increases porosity for both cube and gyroid samples. With porosity greater than 50% for all types, samples are in agreement with desired porosity range of natural bone, making them suitable for tissue ingrowth. This makes all designs suitable for implant application. With increase in pore size of porous structure Young’s modulus of structure decreases for cubical and gyroid structures. In comparison to elastic modulus of natural bone, cube porous structures with pore size 0.3–0.5 mm are suitable for implant application, whereas, for gyroid structure, all samples are suitable for application to implants. Yield strength analysis of porous samples show that yield strength decreases with increase in pore size of scaffold. Comparison to bone yield strength value depicts that, for both cubical and gyroid unit cell types, only structures with pore size of 0.3 mm show similar properties with that of bone. Comparison of all analyzed properties of porous structures with bone properties concludes that cubical and gyroid porous scaffold with pore size 0.3 mm, manufactured with titanium alloy Ti6Al4V, can achieve mechanical properties similar to natural bone. These porous designs can be used to produce porous artificial implants with bone properties and can be reliably manufactured by additive manufacturing. The ultimate goal of this evolution is to produce better performing prostheses with increased mechanical stability and reliability.

## Figures and Tables

**Table 1:** Mechanical Properties of Ti6Al4V [15].

Material	Ti6Al4V alloys
Manufacturing process	Selective Laser Melting
Density	4.41 g/cm^3^
Young’s modulus	109 ± 2.1 GPa
Poisson’s ratio	0.3
Strain life parameter	8.8 ± 0.6%
Tensile Yield Strength	1098 ± 15 MPa
Ultimate Tensile Strength	1237 ± 13 MPa
Compressive Yield Strength	960 MPa
Ultimate Compressive Strength	1000 MPa

**Table 2:** Comparison of analyzed properties for cube and gyroid porous samples.

Sample	Pore Size (µm)		Porosity (%)		Young’s Modulus (GPa)		Yield Strength (MPa)	
	Nominal	Actual	Nominal	Actual	Simulation	Experiment	Simulation	Experiment
Cube 0.3	300	264.6 ± 19.3	71.35	57.48 ± 2.6	16.3	14.586 ± 0.51	33	28.1 ± 1.58
Cube 0.4	400	365.2 ± 26.6	80.5	70.99 ± 0.7	10.2	8.316 ± 0.34	20	16.02 ± 0.57
Cube 0.5	500	455.4 ± 28.8	82.69	73.11 ± 3.4	8.9	7.116 ± 0.25	18	14.71 ± 0.23
Cube 0.6	600	564.6 ± 23.2	88.38	79.36 ± 4.4	4.6	3.688 ± 0.28	12	10.79 ± 0.28
Gyroid 0.3	300	286 ± 12.3	70.2	70.99 ± 9.3	13	10.604 ± 0.28	26	22.44 ± 0.46
Gyroid 0.4	400	372.2 ± 23.4	74.8	71.00 ± 9.3	9.9	8.775 ± 0.52	20	18.44 ± 0.39
Gyroid 0.5	500	471.6 ± 14.5	78.2	77.21 ± 7.0	8	7.801 ± 0.23	16	13.43 ± 0.24
Gyroid 0.6	600	558.4 ± 23.2	81.1	77.86 ± 8.2	6.3	5.606 ± 0.36	13	11.25 ± 0.31
